# Optical Controlled Terahertz Modulator Based on Tungsten Disulfide Nanosheet

**DOI:** 10.1038/s41598-017-13864-5

**Published:** 2017-11-01

**Authors:** Zhiyuan Fan, Zhaoxin Geng, Xiaoqin Lv, Yue Su, Yuping Yang, Jian Liu, Hongda Chen

**Affiliations:** 1 State Key Laboratory of Integrated Optoelectronics, Institute of Semiconductors, Chinese Academy of Sciences, Beijing, 10083 China; 2 School of Information Engineering, Minzu University of China, Beijing, China; 3 College of Science, Minzu University of China, Beijing, 100081 China; 4 State Key Laboratory of Superlattices and Microstructures, Institute of Semiconductors, Chinese Academy of Sciences, Beijing, 10083 China; 5 College of Materials Science and Opto-Electronic Technology, University of Chinese Academy of Science, Beijing, 100049 China

## Abstract

The terahertz (THz) modulator, which will be applied in next-generation wireless communication, is a key device in a THz communication system. Current THz modulators based on traditional semiconductors and metamaterials have limited modulation depth or modulation range. Therefore, a THz modulator based on annealed tungsten disulfide (WS_2_, p-type) and high-resistivity silicon (n-type) is demonstrated. Pumped by a laser, the modulator presents a laser power-dependent modulation effect. Ranging from 0.25 to 2 THz, the modulation depth reaches 99% when the pumping laser is 2.59 W/cm^2^. The modulator works because the p-n heterojunction can separate and limit carriers to change the conductivity of the device, which results in a modulation of the THz wave. The wide band gap of WS_2_ can promote the separation and limitation of carriers to obtain a larger modulation depth, which provides a new direction for choosing new materials and new structures to fabricate a better THz modulator.

## Introduction

In past decades, the requirements for higher speed and broader bandwidth communication systems have continuously increased. Recently, the terahertz (THz) wave, with a wavelength of 0.03–3 mm, has attracted researchers’ attention due to its various advantages in wireless communication. Basic devices for THz wireless communication, such as THz wave sources, THz wave detectors and THz wave modulators, have been investigated by many research groups. Different mechanisms and devices for THz modulators have also been reported^1–5^. THz modulators are based on traditional materials, such as silicon and gallium arsenide (GaAs), and they always have a small modulation depth because of the recombination of carriers in materials^2^. To overcome the small modulation depth, human-made metal micro-nanostructured materials, namely, metamaterials, have been used for THz modulation because metamaterials have many unique optical and electrical properties. For example, in 2006, Chen *et al*. first fabricated a THz modulator based on a metamaterial, which enabled the modulation of THz transmission by 50%^6^. Since then, many studies using THz modulators based on metamaterials have been published^7–9^. These devices provide tuneable, nonlinear and high modulation effects. However, these THz modulators usually work in a specific frequency range that depends on the nanostructure of metamaterials. To achieve both large modulation depth and a wide working range, researchers have sought new materials and structures for THz modulators.

Recently, with the devolvement of two-dimensional (2D) materials, such as graphene and transition metal dichalcogenides (TMDs), some researchers have begun to combine 2D materials with metamaterials to fabricate THz modulators. Ju *et al*. used graphene micro-ribbon arrays to absorb THz waves through plasmon resonance^10^. Graphene metamaterial demonstrated a strong plasmon couple to THz radiation, absorbing over 13% at the plasmon resonance. Because of the complexity in fabricating metamaterials, depositing or transferring 2D materials directly onto a substrate has become another way to fabricate THz modulators. Weis *et al*. first formed a THz modulator based on graphene pumping by a femtosecond laser pulse source^11^. However, it is not practical to use femtosecond lasers in THz communication for commercial applications. Li *et al*. reported a dual control method for THz modulation based on a graphene-silicon hybrid diode^12^. Continuous wave (CW) source and bias voltage were applied to control the electron transfer between graphene and silicon. Chen *et al*. used another 2D material, molybdenum disulfide (MoS_2_), to build an ultrasensitive THz modulator under pumping by a CW source^13^. Unlike graphene, Mo_2_ does not have ultrahigh electronic mobility. The working principle of Mo_2_-based modulators lies in the band structure of MoS_2_. To improve the modulation ability of 2D materials, Cao *et al*. formed an optically tuned THz modulator based on annealed multilayer MoS_2_^14^. After an annealing treatment, the MoS_2_ was p-type doped, which effectively enhanced the modulation depth. These studies indicated that 2D materials are promising materials for the THz regime and for THz modulators.

Tungsten disulfide (WS_2_), as another member of the TMDs, has a similar structure and properties to MoS_2_. Each WS_2_ monolayer contains a single layer of tungsten atoms sandwiched by two sheets of sulfur atoms in a trigonal prismatic coordination. Like other TMDs, WS_2_ exhibits a layer number-dependent band gap. Monolayer WS_2_ has a direct band gap of ~2 eV, while multilayer WS_2_ and bulk WS_2_ have an indirect band gap range from ~1.8 to ~1.3 eV^15^. However, WS_2_ has some distinct advantages over MoS_2_ and other 2D materials. WS_2_ has superior thermal and oxidative stability compared to MoS_2_^16,17^. Furthermore, it only has a weak impurity band, which brings it much higher on/off ratios and much larger current in transistors and optoelectronic devices^18,19^. These excellent properties indicate that WS_2_ could be used as a new material to fabricate various electronic and optoelectronic devices. Therefore, in this paper, WS_2_ was ¬first applied in a THz modulator. The results of the experiment demonstrate that the THz modulator based on annealed WS_2_ and silicon has a rather large modulation depth when pumping by a CW source. Compared with reported results of a THz modulator based on MoS_2_ and graphene, the WS_2_-based device presents a competitive modulation efficiency under similar conditions. Importantly, the working mechanisms of the WS_2_- and MoS_2_-based modulators are discussed in detail. Based on our analytical model and experiment results, a clear direction for designing more effective THz modulators is noted.

## Results and Discussion

### Preparation of the THz modulator based on WS2/Si heterostructure

**Figure 1:**
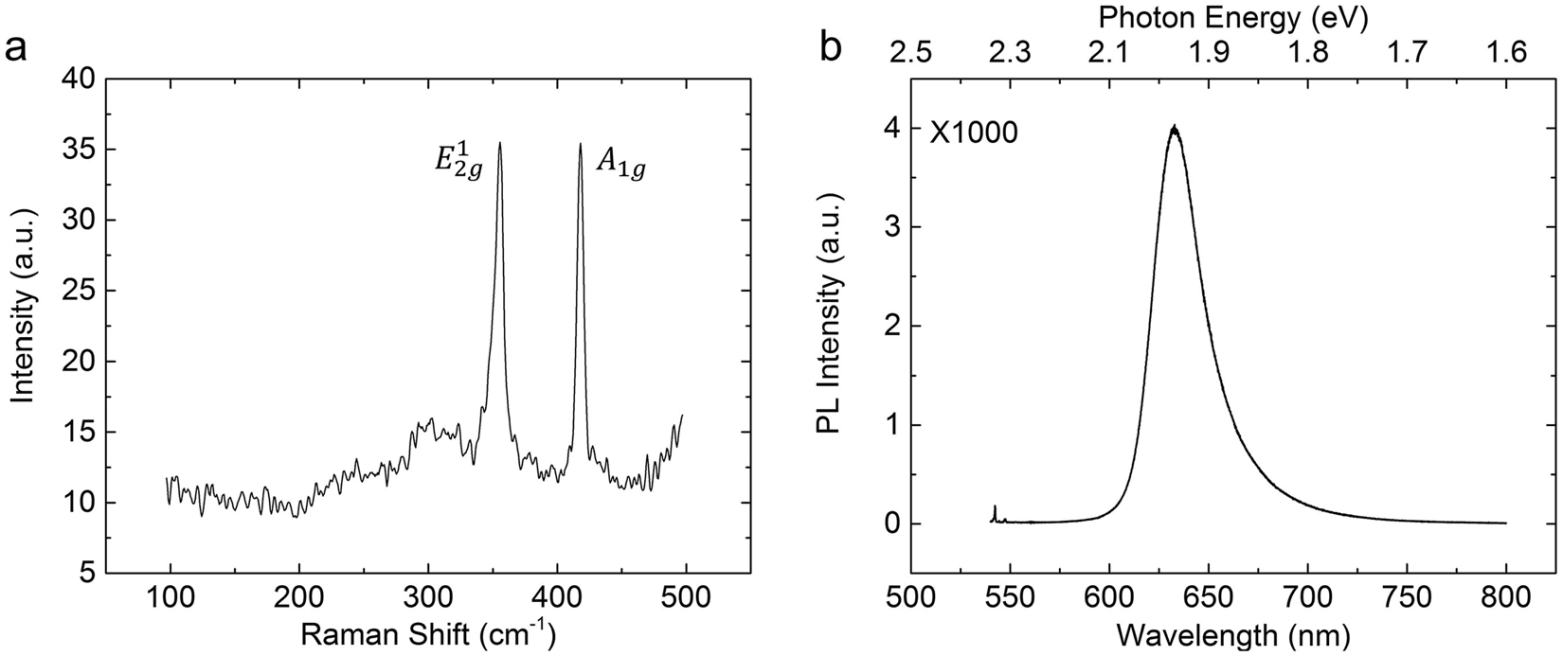
Characteristics of WS_2_. (**a**) Raman and (**b**) photoluminescence spectra of WS_2_ on a high-resistivity silicon substrate.

THz modulators work by changing the conductivity of a device, which is primarily determined by the concentration of free carriers in the device. To achieve a significant change in the concentration of free carriers before and after illumination by a laser, lightly doped silicon and materials with long carrier lives, such as germanium, were used in previous work^14,20^. Hence, the substrate in this work was lightly n-type doped, 1-mm-thick high-resistivity (HR, resistivity ρ > 5000Ω∙cm) silicon. WS_2_ was grown on a sapphire substrate by chemical vapor deposition (CVD). It was later transferred to the substrate with the PMMA-transfer method. The area of WS_2_ grown on the substrate was 1 cm × 1 cm. To identify whether the WS_2_ thin film existed on the substrate, Raman spectroscopy and photoluminescence were used after transfer. The Raman spectrum, after excitation by a 488-nm laser, is shown in Fig. 1a. Two primary peaks, whose positions were located at 355.5 and 418 cm^−1^, agree with the results of several reports^21–23^. In addition, we measured the photoluminescence (PL) spectrum of WS_2_ to acquire more information about the sample. The results of the PL spectrum indicated that the WS_2_ had a band gap of 2.0 eV (Fig. 1b). The results from the Raman and PL spectra indicated that the WS_2_ thin film was successfully transferred to the substrate^21,24^.

### Characterization of the THz modulator based on WS_2_/Si heterostructure

**Figure 2:**
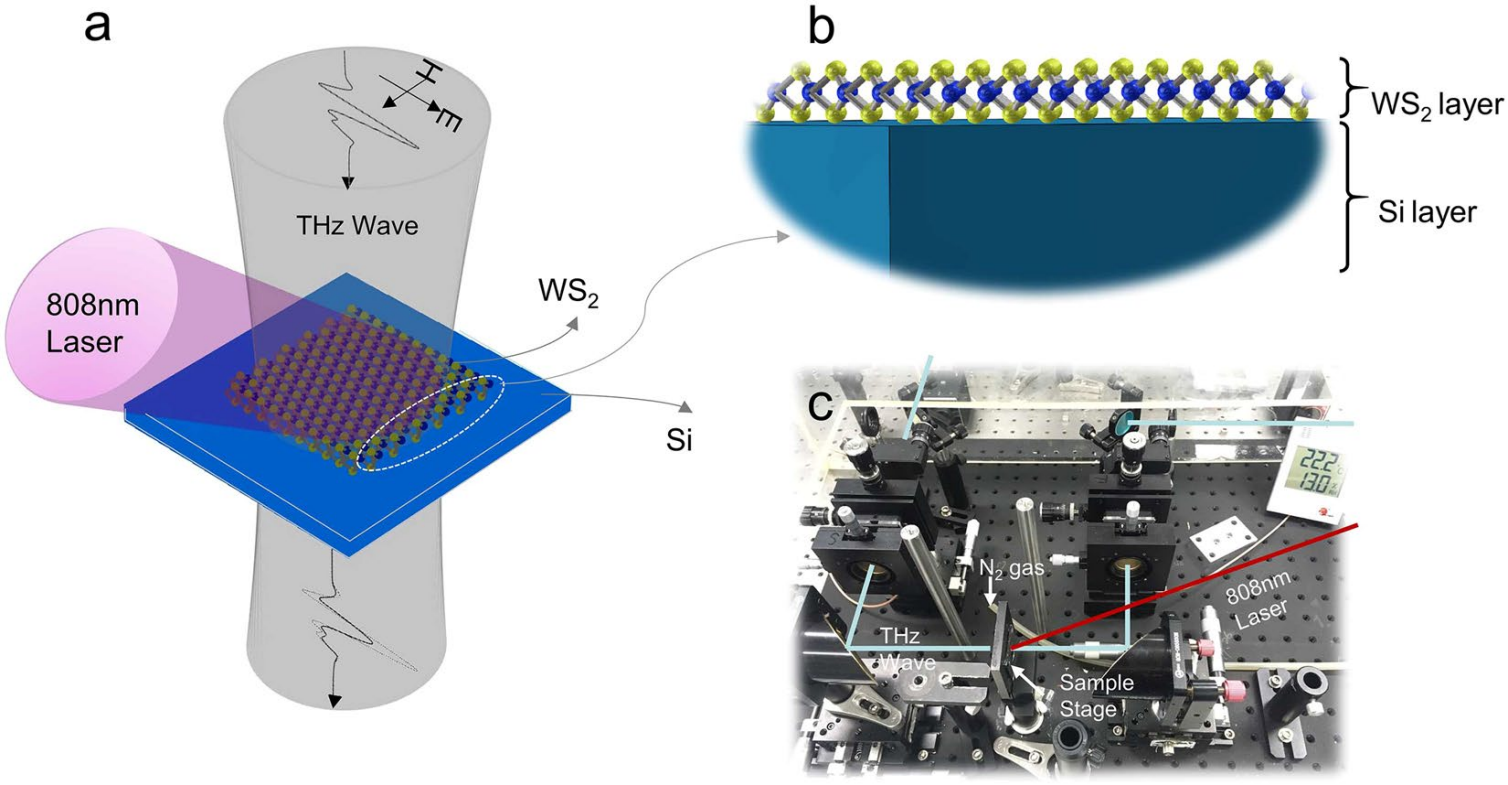
Structure of modulator and testing setup. (**a**) Structure of the THz modulator based on WS_2_ and silicon. (**b**) Layer structure of the THz modulator based on WS_2_ and silicon (**c**) photoluminescence spectra of WS_2_ on a high-resistivity silicon substrate.

The schematic of the final device and measurements are shown in Fig. 2a. All experiments were conducted on a THz time-domain spectroscopy (THz-TDS) system (Fig. 2b). The diameter of the THz beam is approximately 8 mm. The CW laser emits an 808-nm light beam, and the area of the light beam is approximately 1.76 cm^2^. During the experiment, a mask was used to limit the laser to illuminating WS_2_ only. Considering the impact of the water, which could strongly absorb the THz wave, a closed chamber filled with nitrogen gas was used to cover all the measurement equipment.

**Figure 3:**
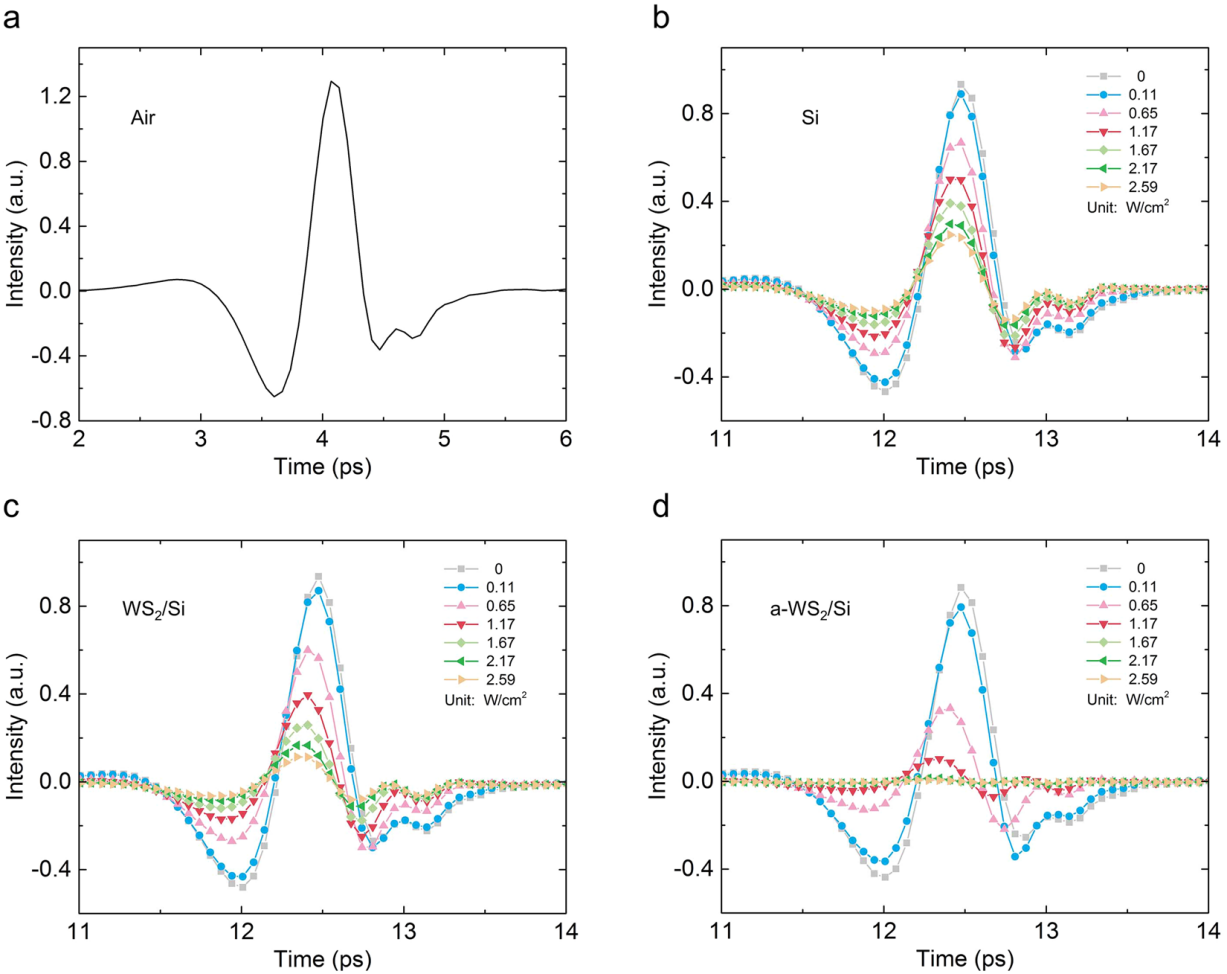
Time-domain intensity of the transmission of the THz signal with different sample under different optical pump power densities. (**a**) Result of the air, (**b**) Result of the HR silicon sample, (**c**) Result of the WS_2_/Si sample and (**d**) Result of the a-WS_2_/Si sample.

The time-domain spectrum of the THz wave was directly measured by the THz-TDS (Fig. 3). The spectrum of air (Fig. 3a) was measured as a reference to account for the influence of environment factors, such as humidity. The spectrum of HR silicon under different pumping laser power densities was measured at the same time as another reference (Fig. 3b). The spectra of HR silicon had a time delay and a height decrease, compared with the spectrum of air. These differences are because the refractive index of silicon is larger than that of air. On the other hand, the shape of these spectra changed little. These results show that there is little absorption in the silicon, and the change of height is mainly due to light reflection.

Figure 3c demonstrates the time-domain intensity of the transmitted THz signal of the WS_2_/Si sample under the same conditions as the measurement on HR silicon. With the increased illumination of the pumping laser on the HR silicon and the WS_2_/Si sample, the transmission intensity of the THz signal gradually decreased. This indicated that the pumping laser could generate electrons in both the HR silicon and the WS_2_/Si samples and these electrons could reflect more THz waves. The intensity of the transmission of the THz signal of the WS_2_/Si sample was clearly smaller than that of the HR silicon, which means the interaction between WS_2_ and silicon can also influence THz transmission.

The MoS_2_ sample can be effectively doped with oxygen and provide numerous holes by annealing in air at the proper temperature^25^. Therefore, WS_2_ received the same treatment, because WS_2_ has a very similar structure to MoS_2_. Hence, the WS_2_/Si sample was annealed in air at 300 °C for 5 hours. The WS_2_/Si sample, after annealing (a-WS_2_/Si sample), was also measured on the THz-TDS system. The time-domain intensity of the transmitted THz signal of the a-WS_2_/Si sample is shown in Fig. 3d. The intensity of the THz signal was significantly reduced after transmitting the a-WS_2_/Si sample, compared with the transmission spectra of the HR silicon and the WS_2_/Si samples. These results illustrate that the annealing treatment could effectively reduce the THz transmission of the sample.

**Figure 4:**
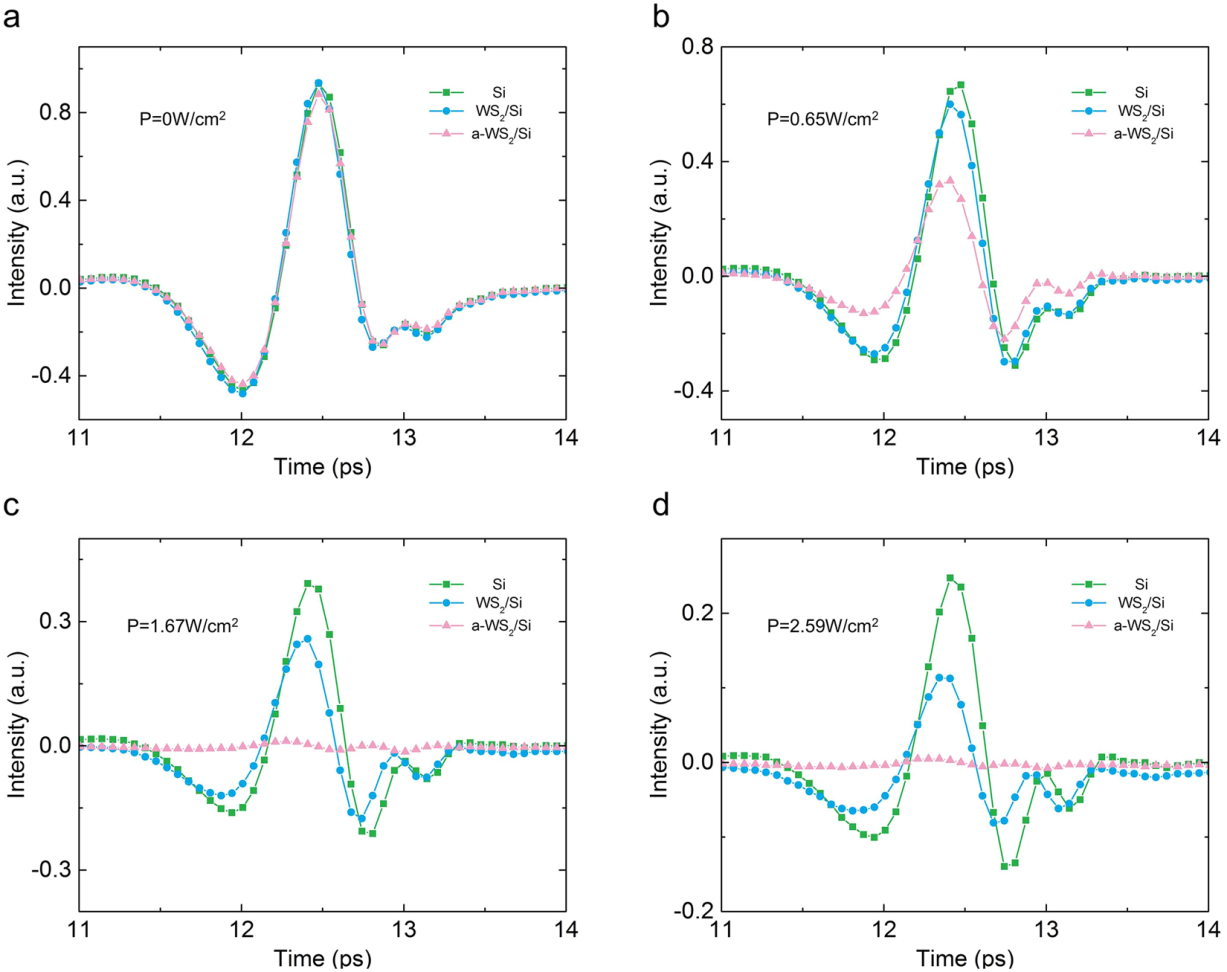
The intensity of transmission of the THz signal by HR silicon, WS_2_/Si and a-WS_2_/Si samples under same optical pump power densities. The power density of the pumping laser was (**a**) 0 W/cm^2^, (**b**) 0.65 W/cm^2^, (**c**) 1.67 W/cm^2^ and (**d**) 2.59 W/cm^2^.

For a clearer observation of the modulation effect for a different sample, the time-domain intensity of the transmission of the THz signal under the same pumping laser power is demonstrated in Fig. 4. Without illumination by the pumping laser, the intensity of the transmission of the THz signal had few differences between the HR silicon, WS_2_/Si and a-WS_2_/Si samples (Fig. 4a), which meant that WS_2_ is almost transparent for the THz wave.

When the power density of the laser was increased to 0.65 W/cm^2^, the WS_2_ and the a-WS_2_/Si samples demonstrated a lower intensity of transmission for the THz signal than that of the HR silicon (Fig. 4b).

When the power density of the laser increased to 1.67 and 2.59 W/cm^2^, the difference in the transmission for the THz signal between the HR silicon and WS_2_/Si increased. In particular, the intensity of the THz signal decreased to almost zero when it was transmitted through the a-WS_2_/Si sample at same time (Fig. 4c,d). The results in Fig. 4 indicate that both the WS_2_/Si and a-WS_2_/Si samples could reflect more THz waves than the HR silicon sample when illuminated by the laser. Specially, a-WS_2_/Si had a lower intensity of transmission for the THz signal under a low pumping power density compared with the results of the WS_2_/Si and HR silicon samples.

**Figure 5:**
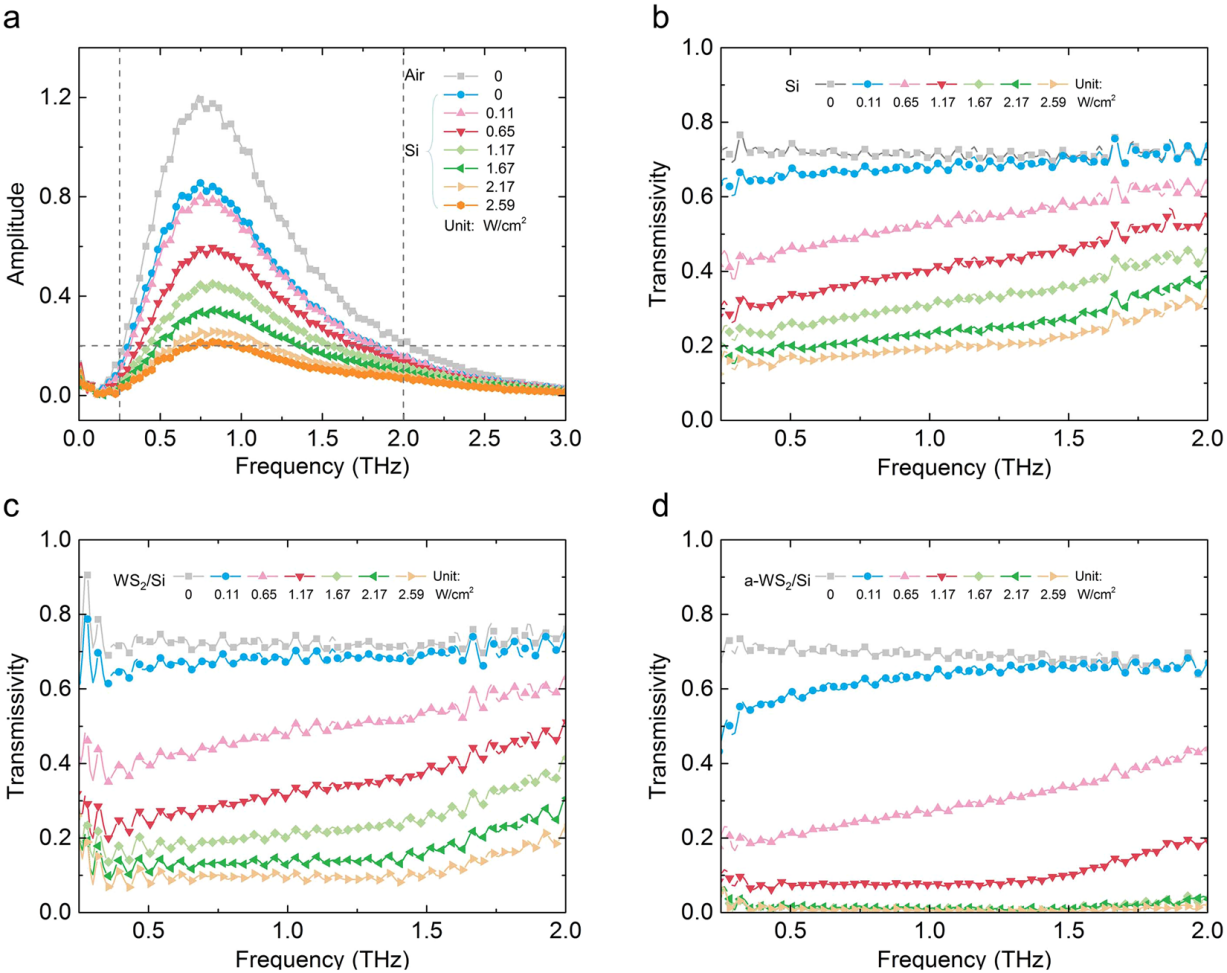
Frequency-domain intensity of THz amplitude and the transmission of the THz signal with different sample under different optical pump power densities. (**a**) Relationship between amplitude and frequency of air and silicon. Relationship between THz transmissivity and frequency of the (**b**) HR silicon, (**c**) WS_2_/Si and (**d**) a-WS_2_/Si samples.

The fast Fourier transfer (FFT) method was applied to transfer data from the time domain into data in the frequency domain. The amplitude of the THz wave acquired from the results of the FFT method is shown in Fig. 5a. The effective part of frequency domain signal is from 0.25 to 2 THz due to limits of the THz generator and detector. THz transmissivity was calculated by Equation 1:

1 $${\boldsymbol{T}}=\frac{{{\boldsymbol{A}}}_{{\boldsymbol{s}}}}{{{\boldsymbol{A}}}_{{\bf{0}}}}$$

where ***A***_***S***_ and ***A***_***0***_ represent the THz wave amplitude after crossing the sample and air, respectively. The relationship between the transmissivity of the samples and the frequency under different pumping laser power densities is shown in Fig. 5b–d. As the power density of the pumping laser increased, the THz transmissivity of all samples decreased. As the frequency increased from 0.25 to 2.0 THz, the transmissivity of the HR silicon sample increased slightly, which indicated that the THz modulator based on HR silicon could not work at high frequencies.

However, for the WS_2_/Si and the a-WS_2_/Si samples, the increase began at 1.5 THz due to the limit of the Drude model, which has been discussed in ref.^8^. These results illustrate that the WS_2_/Si and a-WS_2_/Si samples could stably work from 0.25 to 1.5 THz.

**Figure 6:**
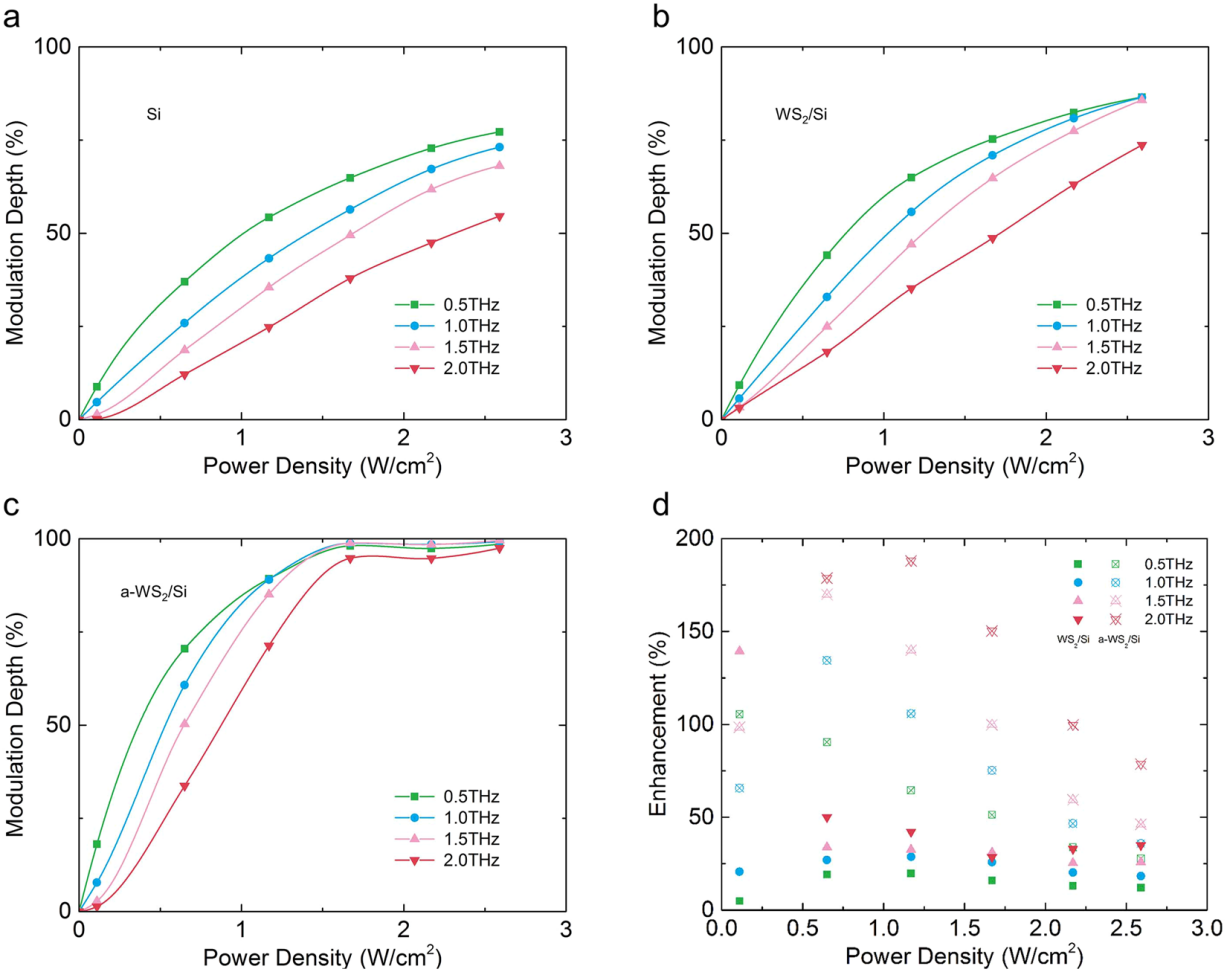
Modulation depth of different samples and the enhancement. (**a**) HR silicon, (**b**) WS_2_/Si and (**c**) a-WS_2_/Si samples under different pumping power densities and at different frequencies. (**d**) Enhancement rate of the WS_2_/Si and the a-WS_2_/Si samples compared with the HR silicon sample.

The lowest THz transmissivity of a-WS_2_/Si sample measured in this work was an average of 0.736%, ranging from 0.25 to 2.0 THz, when the pumping laser power was 2.59 W/cm^2^. A relatively low THz transmissivity was reached, 1.385%, when the pumping laser power was 1.67 W/cm^2^. The modulation depth was also calculated for a clear comparison with other THz modulators. The computational formula for modulation depth is Equation 2:

2 $${\boldsymbol{M}}=| \frac{({{\boldsymbol{T}}}_{{\boldsymbol{i}}}\,-\,{{\boldsymbol{T}}}_{{\bf{0}}})}{{{\boldsymbol{T}}}_{{\bf{0}}}}| $$

where ***T***_***i***_ and ***T***_***0***_ are the THz transmissivity with and without pumping light, respectively. The modulation depth of the HR silicon, WS_2_ and a-WS_2_/Si samples is demonstrated in Fig. 6a–c. At different frequencies, the modulation depth of the same sample at the same pumping power density is different. The results (Fig. 6) show that the HR silicon and WS_2_/Si samples declined significantly in modulation depth when the frequency increased from 0.5 to 2.0 THz under the same pumping power density. However, the a-WS_2_/Si sample had a small decline of modulation depth when the frequency increased. In particular, when the pumping power density was larger than 1.67 W/cm^2^, the modulation depth of the a-WS_2_/Si sample declined little compared with that of the HR silicon and the WS_2_/Si samples. In Fig. 6d, we demonstrated the enhancement rates of the WS_2_/Si and the a-WS_2_/Si samples compared with that of the HR silicon sample.

Removing data caused by noise, Fig. 6d demonstrates that the a-WS_2_/Si sample had a 188% enhancement (E) compared with that of the HR silicon sample, which was calculated by Equation 3:

3 $${\boldsymbol{E}}=| \frac{({{\boldsymbol{M}}}_{{\boldsymbol{S}}}\,-\,{{\boldsymbol{M}}}_{{\boldsymbol{Si}}})}{{{\boldsymbol{M}}}_{{\boldsymbol{Si}}}}| $$

where ***M***_***S***_ represents the modulation depth of the WS_2_/Si and a-WS_2_/Si samples; and ***M***_***Si***_ represents the modulation depth of the HR silicon. The calculation was carried out when the data had the same frequency and power density.

## Discussion

The conductivity of materials affects the transmission of the THz wave^13,14,26^. Hence, the modulation depth of the THz modulator based on the WS_2_-silicon heterojunction is mainly determined by the conductivity of the device, which changes with the behaviour of the carriers. Therefore, analysing and understanding the behaviours of the carriers is the key point in understanding the mechanism by which THz modulators work.

During the experiment, the transmissivity of all samples declined when the pumping laser illuminated samples. This indicated that the conductivity of the devices changed because free carriers were generated in the materials after absorbing photons. Since the laser is 808 nm (1.53 eV), most light transmitted the WS_2_ (*E*_*g*_ = 2 eV) and was absorbed by silicon (*E*_*g*_ = 1.12 eV). As silicon absorbed most of the light, photogenerated carriers were mainly generated in silicon. Without WS_2_, these photogenerated carriers could not exist in silicon for very long due to recombination, which limited the number of electrons, as well as changes in the conductivity of the device. An effective way to break this limitation is separating electrons and holes into different areas to prevent collision. The built-in electric field in the WS_2_-silicon heterojunction could promote the separation, and this is a key factor in WS_2_-silicon-based THz modulators.

**Figure 7:**
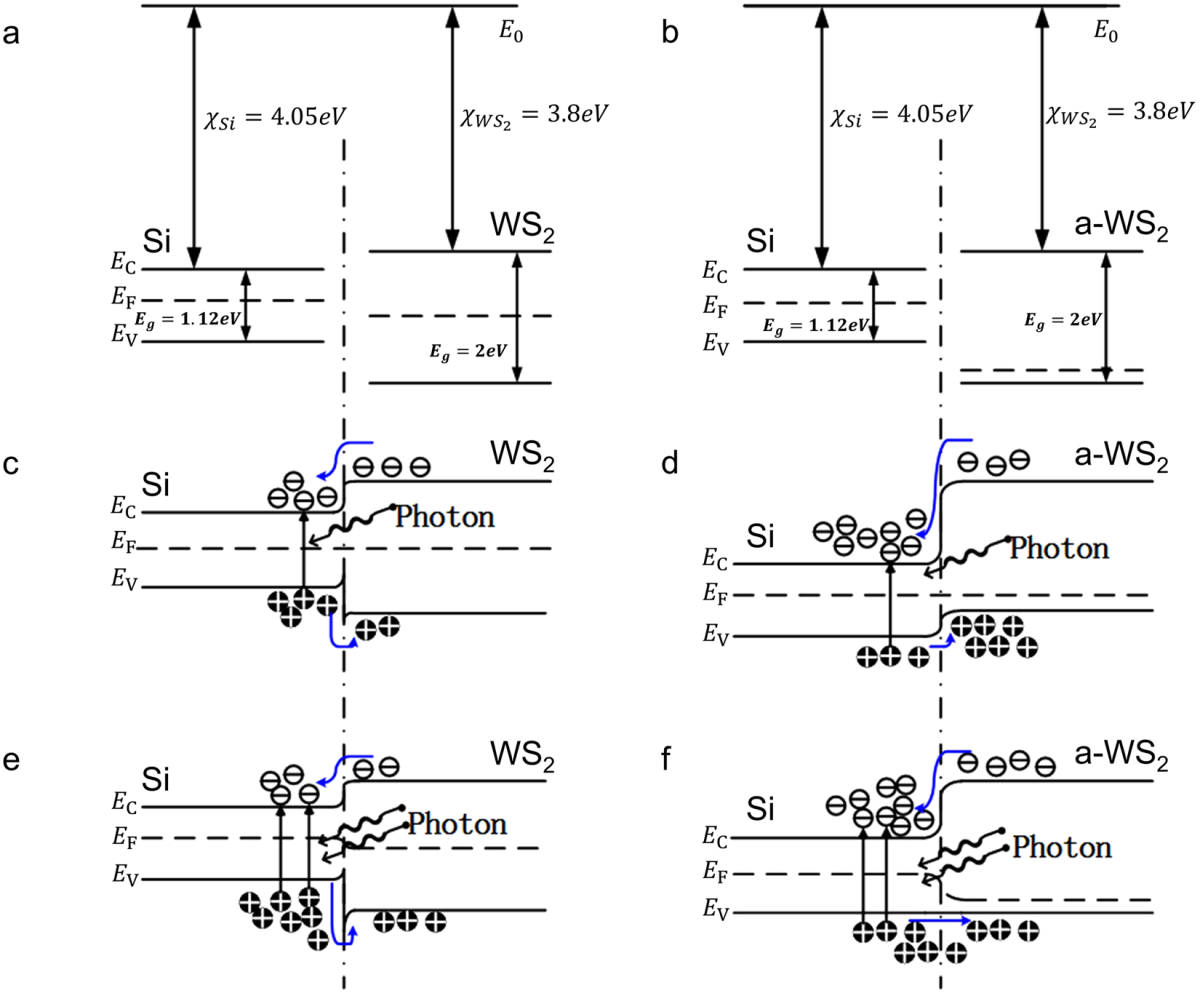
Energy band structure of different samples. (**a**) Energy band structure of the high-resistivity silicon, WS_2_ and (**b**) a-WS_2_ before contact. Band structure of the WS_2_-Si sample before annealing under (**c**) low and (**e**) high pumping laser power. Band structure of the WS_2_-Si sample after annealing under (**d**) low and (**f**) high pumping laser power.

In the WS_2_-silicon sample that was not annealed, both WS_2_ and silicon have Fermi levels near the centre of the bandgap with slight difference (Fig. 7a). Therefore, a I-type heterojunction was formed when they came into contact (Figs. 4e and 7c). Electrons and holes generated in silicon cannot be effectively separated and are easily recombined due to collision. Recombination between the electron and the hole leads to a slight change in the concentration of free carriers as well as a slight change in the conductivity of a device. After an annealing treatment, WS_2_ became a p-type semiconductor and formed a II-type heterojunction with the HR silicon (Fig. 7b). In the II-type heterojunction, the potential barrier for electron V_D_ was calculated by Equation 4:

4 $${{\boldsymbol{V}}}_{{\boldsymbol{D}}}=| ({E}_{0}\,-\,{E}_{F(Si)})\,-\,({E}_{0}\,-\,{E}_{F(W{S}_{2})})| $$

where the *E*_0_ represents the vacuum electron energy. *E*_*F*(*Si*)_ and ${E}_{F(W{S}_{2})}$ represent the fermi level in silicon and WS_2_ respectively. The annealing treatment lower the fermi level in WS_2_ which leading to a larger *V*_*D*_ as well as a larger built-in electric field. Under the electrostatic force of the built-in electric field, photogenerated electrons were transferred from WS_2_ to silicon, or in other words, they were limited in silicon (Figs 4f and 7d). On the other hand, holes moved in the opposite direction and combined in WS_2_. Therefore, electrons and holes were separated and limited in different areas of the device. The separation in space made it hard for the electrons and holes to meet each other and collide. The low collision probability resulted in the long life time of the free carriers. As more electrons were generated and localized in the silicon, the conductivity of the silicon was enhanced. Based on the theory of electromagnetic fields, the THz wave would be reflected by materials with high conductivity. Therefore, a THz modulator based on a WS_2_-silicon heterojunction was demonstrated.

THz modulators based on WS_2_/Si and MoS_2_/Si have similar structures. Hence, the working mechanism of these modulators is also similar. A key factor of the working mechanism lies in the separation of free carriers, which is promoted by the built-in electric field. However, the results show that the WS_2_-silicon sample has a larger modulation depth than the MoS_2_-silicon sample. The difference in the band gap between WS_2_ and MoS_2_ is a key point to explain this result. When two materials come into contact, a potential barrier, which is mainly determined by the band structure of two materials, is formed along with a built-in electric field. The potential barrier can influence the diffusion of carriers, while the built-in electric field mainly influences the drift of carriers. The height of the potential barrier, which is determined by the difference in the conduction band minimum and valance band maximum of the materials, influences whether the carriers can diffuse between the two materials. Unlike carrier drift under the force of the built-in electric field, carriers diffusing from areas of high concentration those with low concentration need to overcome the force of the electric field and potential barriers. The number of electrons in silicon will increase as the power of the laser increases. The force of the electric field will decrease due to electron accumulation; the potential barrier will also decrease due to changes in the band structure (Fig. 7); and the diffusion rate of electrons will increase due to the larger difference in the electron concentration. For holes, the drifting rate is larger than the diffusion rate because the difference in the concentration of the holes is rather small. These discussions indicated that there was a limitation in the modulation depth that was decided by the balance between the built-in electric field, electron concentration and potential barrier. Both MoS_2_ and WS_2_ could hardly form the III-type heterojunction with silicon. Therefore, the potential barrier should meet the Formula 5:

5 $${V}_{D}\ll {E}_{g(X{S}_{2})}$$

where ${E}_{g(X{S}_{2})}$ represents the band gap of MoS_2_ or WS_2_. Since ${E}_{g(W{S}_{2})}$ is larger than ${E}_{g(Mo{S}_{2})}$, WS_2_-silicon heterojunction could achieve a higher potential barrier than MoS_2_-silicon heterojunction. The larger potential barrier could effectively prevent electrons from diffusing from silicon to WS_2_ which leading to a larger modulation depth when illuminated by a high-power pumping laser.

The Table 1 listed the results of THz modulator based on TMDs, graphene and other material-silicon heterostructure. Comparing to C_60_ and AlClPc (Chloride aluminium phthalocyanine), WS_2_ is thinner, more stable and compatibility with silicon process. Comparing to graphene, WS_2_ demonstrated competitive modulation depth while needed lower motivation power. Among the TMDs, WS_2_ demonstrated larger modulation depth as analysed above.

**Table 1:** Some results of reported THz modulator based on materials-silicon heterostructure.

Year	Materials	Treatment	Modulation Depth	Motivative Laser	Ref
—	WS_2_	CVD, PMMA-transfer and annealing	99%	808 nm, 2.59 W/cm^2^	This work
2012	Graphene	CVD and thermal release tape transfer	99%	780 nm, 1.6×10 ^4^mW/cm^2^	11
2014	C_60_	Thermal evaporation deposited, annealing	98%	785 nm, 955 mW/cm^2^	27
2015	AlClPc	Thermal evaporation deposited	99%	450 nm, 1.57 mW/cm^2^	3
2015	Graphene	CVD, PMMA-transfer	83%	532 nm, 420 mW; −4V bias voltage	12
2016	MoS_2_	CVD, PMMA-transfer and annealing	96%	808 nm, 4.56 W	14
2016	MoS_2_	CVD	75%	532 nm, 0.24 W/cm^2^	13

## Methods

### WS_2_ Transfer

The 1 cm × 1 cm WS_2_ thin film was grown on a sapphire substrate using the CVD method. PMMA/anisole solution was dropped on the surface and covered all the WS_2_. After the anisole in the solution evaporated, a NaOH solution (~3 mol/L) was used to etch the sapphire substrate. WS_2_ and PMMA were separated from the sapphire substrate. The WS_2_/PMMA film was transferred to a high-resistivity silicon substrate whose size was 1.5 cm × 1.5 cm. Finally, the PMMA was dissolved by acetone.

### Annealing treatment

The WS_2_/Si sample was annealed on air. The heating rate was set as 10 °C/min. The annealing temperature was set at 300 °C and the annealing time was 5 hours. After being heated, the sample was cooled naturally.

### THz measurement

The sample was measured using a THz time-domain spectroscope system. N_2_ gas was used to protect the sample and maintain dry conditions during the measurement. The avenge relative humidity rate during measurements was 9%.

## Conclusions

In conclusion, a THz modulator based on a WS_2_-Si heterostructure was presented in this work. The modulation effect could be modified by changing the power of pumping laser. After being annealed in air, the THz modulation depth of the modulator was significantly enhanced. The largest modulation depth reached 99%, ranging from 0.25 to 2 THz when the power of pumping laser was 2.59 W/cm^2^. An analytical model was proposed to explain the large modulation depth of the THz modulator based on the annealed WS_2_-silicon heterojunction. WS_2_ became a p-type semiconductor after the annealing treatment and formed a p-n heterojunction with the n-type HR silicon. The p-n heterojunction could separate electrons and holes quickly and effectively. On the other hand, WS_2_ has a wide band gap that forms a high potential barrier at the interface, which could prevent electrons from diffusing to WS_2_. Therefore, a rather high conductivity of the sample was obtained, which resulted in a larger THz modulation depth. These results indicate that WS_2_ is a promising material for THz modulators. Based on our analytical model, 2D materials with wider band gaps, such as boron nitride (BN), and 2D material heterojunctions, which can separate carriers quickly and effectively, might be novel and promising choices for THz modulators.
